# Biological Context–Informed and Population‐Stratified Strategies Improve Genetic Diagnosis of *CCDC22*‐Related Disorder

**DOI:** 10.1155/genr/8137770

**Published:** 2026-06-24

**Authors:** Pei-Qi Zhang, Peng-Yu Wang, Si-Qi Zhang, Yan Ding, Wen-Hui Liu, Yao Wang, Kai Peng, Hua Li, Sheng Luo, Heng Meng, Jing Guo

**Affiliations:** ^1^ Epilepsy Center, Guangdong Sanjiu Brain Hospital, Guangzhou, Guangdong, China; ^2^ Institute of Neuroscience, Key Laboratory of Neurogenetics and Channelopathies of Guangdong Province and the Ministry of Education of China, The Second Affiliated Hospital, Guangzhou Medical University, Guangzhou, Guangdong, China, gzhmc.edu.cn; ^3^ Department of Neurology, The First Affiliated Hospital & Clinical Neuroscience Institute of Jinan University, Guangzhou, Guangdong, China

**Keywords:** *CCDC22*, ClinPred, *in silico* tools, MetaRNN, missense variant, optimizing genetic diagnosis

## Abstract

**Background:**

The *CCDC22* gene is a key regulator of endosomal trafficking and NF‐κB signaling, and its dysfunction is implicated in a spectrum of X‐linked neurodevelopmental disorders, including Ritscher–Schinzel syndrome 2, intellectual disability, and epilepsy. Despite the identification of *CCDC22* variants having increased, a comprehensive biological characterization, including its spatiotemporal expression and functional molecular networks, remains to be systematically delineated. Furthermore, the rarity of *CCDC22* variants created a significant challenge in distinguishing their pathogenicity. This study aimed to explore the integration of biological context‐informed and clinical genetic analysis workflows and to perform an exploratory evaluation of prediction algorithms to better optimize the genetic diagnosis process for *CCDC22*.

**Methods:**

Multilevel biological analysis was performed to evaluate the spatiotemporal expression patterns of *CCDC22* across developmental stages. A protein–protein interaction network was constructed to identify key functional modules and pathway enrichments. Additionally, an expert‐classified dataset of *CCDC22* missense variants was utilized to conduct an exploratory performance evaluation of twenty prediction algorithms, specifically assessing the effect of filtering out variants observed as population hemizygotes.

**Result:**

*CCDC22* exhibits distinct spatiotemporal expression dynamics and chromatin accessibility patterns closely associated with neurodevelopmental processes. PPI and functional enrichment analyses highlighted its core involvement in endocytic recycling and vesicle transport. In the exploratory missense variant evaluation, meta‐predictors, notably ClinPred and MetaRNN, demonstrated the highest predictive potential within this limited cohort. After filtering out variants observed as hemizygotes in the gnomAD database, most of the algorithms’ performance improved in distinguishing the pathogenicity of variants and genetic diagnosis. ClinPred, M‐CAP, MetaRNN, and SIFT achieved the highest balanced accuracy (84.6%, 80.8%, 78.7%, and 76.1%, respectively). ClinPred, M‐CAP, and MetaRNN achieved the highest AUC value (> 0.9).

**Conclusion:**

This study delineates the spatiotemporal and functional molecular network of *CCDC22* in neurodevelopment. A combination of population‐based strategy and prediction enhanced the performance of most algorithms. ClinPred and MetaRNN showed higher predictive potential. This study may provide insights into the evaluation of variants in *CCDC22*‐related diseases.

## 1. Introduction

The approach to investigating Mendelian disorders has transformed over the past few decades, evolving from traditional linkage analysis to genome‐wide high‐throughput technologies [1]. This paradigm shift provides a opportunity for understanding X‐linked disorders, which often present diagnostic conundrums due to their sex‐biased inheritance (hemizygosity in males) and broad phenotypic heterogeneity [2]. Among the numerous X‐linked genes, *CCDC22* (coiled‐coil domain‐containing protein 22) has emerged as a crucial gene implicated in a spectrum of X‐linked neurodevelopmental disorders (NDDs). Located on chromosome Xp11.23, *CCDC22* acts as a core component of the COMMD/CCDC22/CCDC93 (CCC) complex, playing essential roles in endosomal protein trafficking and the regulation of NF‐κB signaling pathways [3–5]. Variants in *CCDC22* have been reported in diverse conditions, including Ritscher–Schinzel syndrome 2 (RTSC2), syndromic and nonsyndromic X‐linked intellectual disability, and X‐linked focal epilepsy [6–8].

Despite its established clinical significance, the comprehensive biological landscape of *CCDC22* during neurodevelopment remains inadequately characterized. To better understand the pathogenic mechanisms underlying *CCDC22*‐related disorders, it is imperative to delineate its functional context. Currently, there is a distinct lack of systematic profiling regarding its dynamic spatiotemporal expression trajectories during brain development, its region‐specific chromatin accessibility, and the broader protein–protein interaction (PPI) networks it orchestrates. Establishing these fundamental biological characteristics is a prerequisite for understanding how structural variations translate into severe neurodevelopmental phenotypes.

The widespread application of next‐generation sequencing (NGS) has led to the discovery of an increasing number of rare *CCDC22* variations. A major diagnostic challenge lies in the fact that a large proportion of these are missense variants. Unlike loss‐of‐function variants, missense variants may alter protein conformation, disrupt specific PPIs, or affect subcellular localization. Because experimental validation of these subtle effects is resource‐heavy and impractical for routine clinical use, medical genetics relies heavily on population databases (e.g., gnomAD) and in silico prediction algorithms to infer pathogenicity [6, 9–11].

However, predicting the pathogenicity of missense variants in ultra‐rare X‐linked genes like *CCDC22* presents unique biological and computational challenges. Many widely used in silico tools are trained on broad, generalized genomic datasets. These generalized training regimes may underrepresent the unique hemizygous nature of X‐linked variants and may not fully capture the specific structural nuances of highly specialized functional regions, such as the coiled‐coil domains central to the *CCDC22* protein architecture. Consequently, applying these general computational tools to ultra‐rare diseases often yields highly volatile performance metrics and reveals critical statistical and biological blind spots. Relying solely on generalized algorithms without considering the specific biological context or stringent population filtering can lead to significant diagnostic ambiguity.

In this study, we analyze the spatiotemporal expression, chromatin accessibility, and PPI molecular networks of *CCDC22* to map its functional biological context in neurodevelopment. We also explored the performance of twenty widely used in silico tools on the prediction of *CCDC22* missense variant pathogenicity. We curated two datasets—disease‐associated variants and benign/likely benign variants—and assessed tool performance against the core metrics: accuracy, sensitivity, specificity, positive predictive value (PPV), negative predictive value (NPV), Matthews correlation coefficient (MCC), F‐score, and area under the receiver operating characteristic curve (AUC). We also tested whether filtering out variants observed as hemizygotes in the gnomAD database would improve the tools’ performance. This study provided a potential baseline for further optimization of the prediction algorithms, highlighting the necessity of integrating biological context with computational and population‐based strategies when evaluating variants in *CCDC22*‐related disorders.

## 2. Materials and Methods

### 2.1. Spatio‐Temporal Expression Pattern of *CCDC22*

The RNA expression of *CCDC22* in various tissues was illustrated using the data from the Genotype‐Tissue Expression (GTEx) dataset (https://www.gtexportal.org/). The data within this dataset predominantly originate from adult individuals. To investigate the spatiotemporal expression pattern of *CCDC22*, the data from the Evo‐devo mammalian organs database (https://apps.kaessmannlab.org/evodevoapp/) and the Brainspan database (https://www.brainspan.org/) were employed. These data from Brainspan encompasses a developmental range from 8 postconceptional weeks to 40 years of age, which was visualized in the Genetic Dependence and Pathogenicity (GD&P) Database (https://www.gdap.org.cn/about/database). To better understand and depict the expression pattern of *CCDC22*, the expression spline was fitted through the application of the locally weighted scatterplot smoothing (LOWESS) algorithm. The scATAC‐seq data of the cerebral organoid were from the ScApeX database.

### 2.2. PPI Network and Enrichment Analysis

The PPI network of *CCDC22* was constructed and analyzed using the STRING database (Version 12.0; https://string-db.org/). Only interactions with a confidence score ≥ 0.7 were retained for downstream analysis. The network type was set to “full STRING network.” The network edges were interpreted based on “evidence.” Active interaction sources included “textmining,” “experiment,” “databases,” “co‐expression,” “neighborhood,” “gene fusion,, and “co‐occurrence”; the maximum number of interactors displayed was set to 500. The PPI network was imported into Cytoscape for visualization, and the interacting proteins were annotated for associated human diseases using the Online Mendelian Inheritance in Man (OMIM) database. Additionally, functional enrichment analyses, including Gene Ontology (GO), Kyoto Encyclopedia of Genes and Genomes (KEGG) pathway, and Reactome pathway analyses, were performed using the interacting proteins.

### 2.3. Variants Collection

To assess the ability of in silico tools to predict the pathogenicity of missense variants in *CCDC22*, variants were split into two groups: disease‐associated variants and controls. Disease‐associated variants were retrieved from the Human Gene Mutation Database (HGMD) and PubMed using the query: *CCDC22* AND (variant OR mutation). These variants were subsequently subjected to a stringent selection process, requiring them to meet the following: (1) the disease diagnosis is supported by sufficient clinical information; (2) the involvement of other known disease‐causing genes is largely excluded; (3) the origin of the variant can be explicitly determined; and (4) the pathogenicity of the variant has been evaluated by experts. Control variants were selected based on the following conditions: (1) the genotype is absent in the non‐neurological control populations of the gnomAD database; (2) the variants are classified as “benign” or “likely benign” in ClinVar; and (3) the minor allele frequency is less than 0.01 in both the gnomAD v2 and v4 datasets to prevent bias in the evaluation of algorithms that rely on population frequencies. The data review cutoff were November 1, 2025.

### 2.4. *In Silico* Prediction

A panel of twenty bioinformatics tools was selected for this study, chosen based on their widespread use and established effectiveness in predicting the functional impact of missense variants. The selection of these tools was based on primary criteria: (1) inclusion in the ACMG variant interpretation guidelines or recommendation by clinical genetics consortia, or availability in major variant annotation databases such as Franklin (franklin.genoox.com) or VarSome (varsome.com); (2) the availability of well‐defined and recommended score thresholds for pathogenicity classification; and (3) the ability to generate a valid score for at least 80% of the variants included in this study. All prediction scores were obtained from the precomputed databases dbNSFP (dbnsfp.org) to ensure reproducibility. These tools are summarized in Table S1 [3–19].

### 2.5. Evaluation of Predictive Performance

The efficacy of the computational tools was evaluated utilizing true positive (TP), true negative (TN), false positive (FP), and false negative (FN) counts, using method descried in our previous studies [20, 21]. These foundational metrics enabled the calculation of accuracy, sensitivity, specificity, positive/negative predictive values (PPV/NPV), F‐score, and the MCC. The F‐score was computed as the harmonic mean of precision and recall. The MCC, serving as a comprehensive measure of binary classification quality, ranges from −1 (complete misclassification) to 1 (perfect prediction). Furthermore, receiver operating characteristic (ROC) curves were generated to evaluate the area under the curve (AUC). Disease‐associated variants were used as the positive group, while missense variants with no relevant phenotypic reports served as negative controls.

### 2.6. Statistical Analysis

All statistical computations were performed using R software (Version 4.5.1). For independent two‐group comparisons, Student’s *t*‐test or the Wilcoxon rank‐sum test was applied, depending on data normality as determined by the Shapiro–Wilk test. A *p*‐value of less than 0.05 was defined as statistically significant.

## 3. Results

### 3.1. Spatio‐Temporal Expression Pattern of *CCDC22*

**FIGURE 1:**
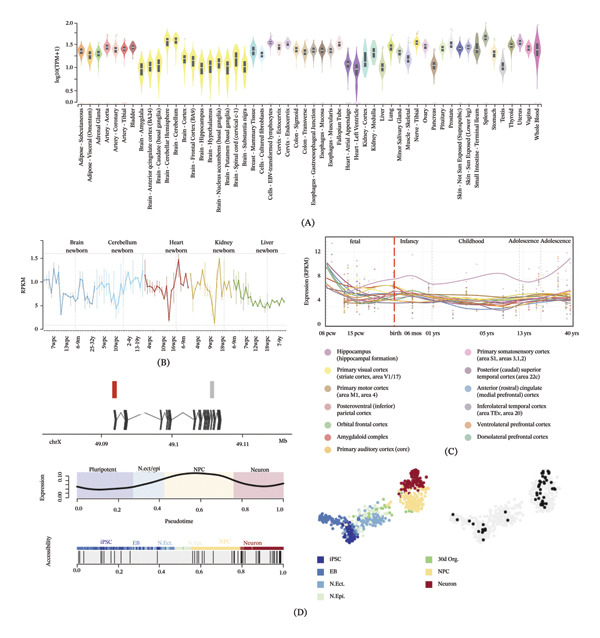
Spatiotemporal expression pattern and chromatin accessibility of *CCDC22*. (A) The RNA expression of *CCDC22* across various adult human tissues. (B) Temporal expression pattern of *CCDC22* across developmental stages in mammalian organs. (C) Spatiotemporal expression pattern of *CCDC22* across multiple brain regions from 8 postconception weeks to 40 years of age. (D) The chromatin accessibility of *CCDC22* during human cortical neurogenesis along the developmental pseudotime from induced pluripotent stem cells to mature neurons.

The spatiotemporal expression pattern of *CCDC22* was systematically investigated to better understand its relationship with the observed clinical phenotypes. The expression data of adult humans showed *CCDC22* is ubiquitously expressed across multiple adult tissues, including the brain cortex, amygdala, hippocampus, and other brain regions (Figure 1A). Developmental data showed high expression in newborns, particularly in the brain and heart (Figure 1B). Consistently, BrainSpan data (from 8 postconception weeks to 40 years) demonstrated highly *CCDC22* expression across multiple brain regions during fetal stages, followed by a general decline throughout childhood, and showed an increasing trend in adulthood (Figure 1C). To further investigate the expression and chromatin accessibility of *CCDC22* during human cortical neurogenesis, the ATAC‐seq data from human brain cortical organoids were analyzed (Figure 1D). Among the neuronal differentiation trajectory from induced pluripotent stem cells (iPSCs) to mature neurons, *CCDC22* was highly expressed in the NPC stage and neuron stage. Chromatin accessibility at the *CCDC22* was elevated in neural progenitor cells and neurons compared with earlier progenitor stages. These findings suggest that *CCDC22* may play a particularly important role during late‐stage neuronal differentiation and maturation in the developing human cortex.

### 3.2. PPI Network and Enrichment Analysis

**FIGURE 2:**
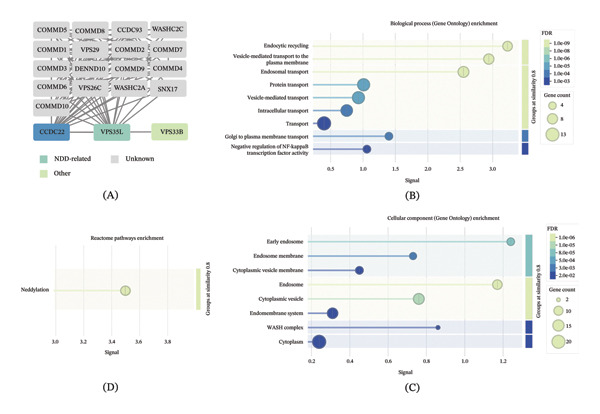
Protein–protein interaction (PPI) network and functional enrichment analysis of *CCDC22*. (A) The PPI network of *CCDC22* and its interacting proteins. Nodes are color‐coded based on their association with human diseases. (B) Top enriched Gene Ontology (GO) terms for biological processes. (C) Top enriched GO terms for cellular components. (D) Significantly enriched Reactome pathway.

To explore the underlying pathogenic mechanism, we investigated the PPI network of the *CCDC22* protein via the STRING database. The PPI network of *CCDC22* included 20 nodes and 130 edges, with a PPI enrichment *p*‐value < 1.0 × 10^−16^ (Figure 2A). Among the interacting proteins, *VPS35L* was identified as an NDD‐related gene, while other interacting proteins, including *COMMD1-10*, *VPS29*, *VPS26C*, *WASHC2A*, *WASHC2C*, *SNX17*, *DENND10*, and *CCDC93*, were associated with unknown disease relevance based on the OMIM database. *VPS33B* was associated with other diseases.

Functional enrichment analyses further revealed the biological roles of the proteins within this network. The top enriched biology process GO terms were predominantly associated with intracellular trafficking pathways, including endocytic recycling, vesicle‐mediated transport to the plasma membrane, endosomal transport, and Golgi‐to‐plasma membrane transport, suggesting that *CCDC22* and its interactors play a central role in vesicle‐mediated protein sorting and membrane trafficking (Figure 2B). The top enriched cellular component GO terms further supported these findings, with interacting proteins predominantly localized to the early endosome, endosome membrane, cytoplasmic vesicle, WASH complex, and the broader endomembrane system (Figure 2C), consistent with its known function as a component of the CCC complex involved in endosomal cargo recycling. Reactome pathway enrichment identified neddylation as a significantly enriched pathway (Figure 2D), suggesting that post‐translational regulation via neddylation may modulate the activity of *CCDC22*‐associated protein complexes. The disruption of *CCDC22* in endosomal recycling and intracellular transport may underlie the neurodevelopmental phenotypes associated with *CCDC22* variants.

### 3.3. Variant Dataset and Phenotypic Characterization

To systematically evaluate the predictive performance of computational tools, a comprehensive dataset including 13 disease‐associated variants and 17 benign/likely benign variants was reviewed from previously published studies on *CCDC22*‐related disorders (Table 1). The disease‐associated variants were associated with diverse clinical phenotypes, including RTSC2[22–27], syndromic X‐linked intellectual disability [23], intellectual disability with dyslipidemia [28], nonsyndromic X‐linked intellectual disability [29], X‐linked focal epilepsy [30], and X‐linked intellectual disability with Ritscher–Schinzel/3C syndrome features [31]. Notably, five disease‐associated variants (p.Arg128Gln, p.Glu239Lys, p.Arg292Cys, p.Arg321Trp, and p.Ala463Gly) were found to have hemizygous occurrences in the non‐neuro controls of the gnomAD database, with allele counts ranging from 1 to 26 individuals. These five variants exhibited phenotypic heterogeneity spanning syndromic to nonsyndromic intellectual disability and RTSC2, suggesting potential incomplete penetrance or variable expressivity. Consequently, parallel analyses were conducted using two datasets: the “before filtered” dataset, including all 13 disease‐associated variants, and the “after filtered” dataset, excluding these five variants with population presence, thereby assessing how gnomAD frequency filtering impacts the discriminative performance of prediction algorithms.

**TABLE 1:** List of likely pathogenic variants of *CCDC22* reported in a previous study.

	Nucleotide	Protein	Hemizygous number in the non‐neuro controls of gnomAD	Variant type	Reported phenotype	Reference
1.	c.49A>G	p.Thr17Ala	—	missense	Ritscher–Schinzel syndrome 2	[22–25]
2.	c.88A>G	p.Thr30Ala	—	missense	Intellectual disability, syndromic X‐linked	[23]
3.	c.112G>A	p.Val38Met	—	missense	Intellectual disability and dyslipidemia	[28]
4.	c.383G>A	p.Arg128Gln	1	missense	Intellectual disability, syndromic X‐linked	[23]
5.	c.700C>T	p.Arg234Cys	0	missense	Ritscher–Schinzel syndrome 2	[26]
6.	c.715G>A	p.Glu239Lys	26	missense	Intellectual disability, syndromic X‐linked	[23]
7.	c.724C>T	p.Arg242Trp	—	missense	X‐linked focal epilepsy	[30]
8.	c.874C>T	p.Arg292Cys	2	missense	Ritscher–Schinzel syndrome 2	[27]
9.	c.961C>T	p.Arg321Trp	2	missense	Intellectual disability, syndromic X‐linked	[23]
10.	c.1388C>G	p.Ala463Gly	1	missense	Intellectual disability, nonsyndromic X‐linked	[29]
11.	c.1486C>T	p.Arg496Cys	—	missense	X‐linked focal epilepsy and focal cortical dysplasia	[30]
12.	c.1670A>G	p.Tyr557Cys	—	missense	Intellectual disability, X‐linked with features of Ritscher–Schinzel/3C syndrome	[29]
13.	c.1858C>T	p.Leu620Phe	—	missense	X‐linked focal epilepsy	[31]

### 3.4. Distributions of the Prediction Scores for the *In Silico* Tools

**FIGURE 3:**
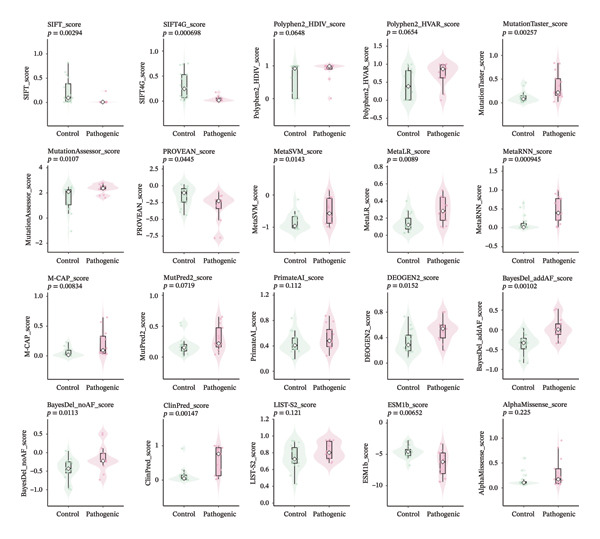
The distribution of in silico prediction scores for *CCDC22* variants before filtered for hemizygous variants in the population. For each algorithm (including SIFT, SIFT4G, Polyphen2_HDIV, Polyphen2_HVAR, MutationTaster, MutationAssessor, PROVEAN, MetaSVM, MetaLR, MetaRNN, M‐CAP, MutPred2, PrimateAI, DEOGEN2, BayesDel_addAF, BayesDel_noAF, ClinPred, LIST‐S2, ESM1b, and AlphaMissense), rawscores are shown for control (green) and disease‐associated variants (or pathogenic, pink) variants. Violin plots depict the score distribution. Higher rankscores indicate more deleterious predictions. Group differences were tested using a two‐sided Wilcoxon rank‐sum test; *p* values are shown in each panel.

To visualize the distributions of predicted scores, the comparison analysis of scores of twenty algorithms between the two groups was performed. In the initial analysis, the majority of prediction tools demonstrated statistically significant differences (*p* < 0.05) between disease‐associated and control variants, including SIFT (*p* = 0.00294), SIFT4G (*p* = 0.000698), MutationTaster (*p* = 0.00257), MutationAssessor (*p* = 0.0107), PROVEAN (*p* = 0.0445), MetaSVM (*p* = 0.0143), MetaLR (*p* = 0.0089), MetaRNN (*p* = 0.000945), M‐CAP (*p* = 0.00834), DEOGEN2 (*p* = 0.0152), BayesDel_addAF (*p* = 0.00102), BayesDel_noAF (*p* = 0.0113), ClinPred (*p* = 0.00147), and ESM1b (*p* = 0.00652). However, several tools were not statistically significant, including Polyphen2_HDIV (*p* = 0.0648), Polyphen2_HVAR (*p* = 0.0654), MutPred2 (*p* = 0.0719), PrimateAI (*p* = 0.112), LIST‐S2 (*p* = 0.121), and AlphaMissense (*p* = 0.225) (Figure 3).

**FIGURE 4:**
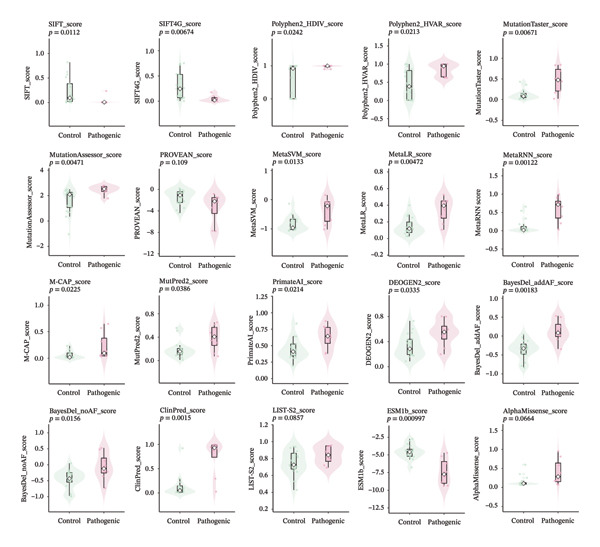
The distribution of in silico prediction scores for *CCDC22* variants after filtering for hemizygous variants in the population. For each algorithm (including SIFT, SIFT4G, Polyphen2_HDIV, Polyphen2_HVAR, MutationTaster, MutationAssessor, PROVEAN, MetaSVM, MetaLR, MetaRNN, M‐CAP, MutPred2, PrimateAI, DEOGEN2, BayesDel_addAF, BayesDel_noAF, ClinPred, LIST‐S2, ESM1b, and AlphaMissense), rawscores are shown for control (green) and disease‐associated variants (or pathogenic, pink) variants. Violin plots depict the score distribution. Higher rankscores indicate more deleterious predictions. Group differences were tested using a two‐sided Wilcoxon rank‐sum test; *p* values are shown in each panel.

To further refine our analysis and reduce potential bias from common population variants, we applied gnomAD allele frequency filtering and re‐evaluated the prediction performance of these tools. Following gnomAD filtering, notable changes in statistical significance were observed: Polyphen2_HDIV (*p* = 0.0242), Polyphen2_HVAR (*p* = 0.0213), MutPred2 (*p* = 0.0386), and PrimateAI (*p* = 0.0214) all showed statistical significance, while PROVEAN (*p* = 0.109) became nonsignificant (Figure 4). The remaining tools largely maintained their statistical significance patterns, with SIFT (*p* = 0.0112), SIFT4G (*p* = 0.00674), MetaRNN (*p* = 0.00122), ClinPred (*p* = 0.0015), and ESM1b (*p* = 0.000997) continuing to show strong discriminative power. LIST‐S2 (*p* = 0.0857) and AlphaMissense (*p* = 0.0664) remained nonsignificant after filtering. The filtering strategy may improve the discriminative capacity of certain prediction algorithms, particularly those that initially showed marginal performance, while most ensemble‐based and machine learning methods maintained robust predictive score distribution.

### 3.5. Performance Evaluation of *In Silico* Tools

**FIGURE 5:**
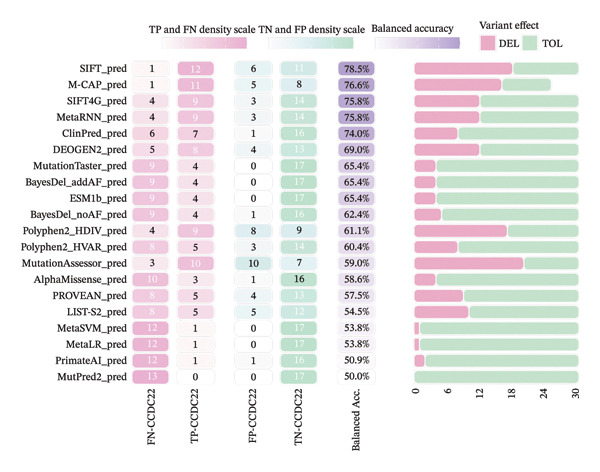
Performance evaluation of algorithms before filtering for hemizygous variants in the population. For each tool, the left heat‐map panel reports the rates of true positives (TP) and false negatives (FN) for disease‐associated variants and true negatives (TN) and false positives (FP) for control variants. The middle column gives the resulting balanced accuracy (balanced acc.), which was used to rank the tools from top to bottom. The right stacked bar shows the proportion of variants each algorithm called deleterious (DEL, red) or tolerated (TOL, blue) using recommended thresholds.

To further evaluate the predictions, we evaluated the confusion matrix of twenty different prediction algorithms, comparing overall classification performance based on metrics of general accuracy, sensitivity (TPR), specificity (TNR), PPV, NPV, MCC, and F1 score (Figure 5, Table 2). SIFT obtained the highest balanced accuracy of 78.5%, followed by M‐CAP (76.6%), SIFT4G and MetaRNN (both 75.8%), and ClinPred (74.0%). Some tools demonstrated discordant performance patterns across different metrics. For example, MutationTaster, BayesDel_addAF, and ESM1b all showed perfect specificity (100%) with severely compromised sensitivity (30.8%), resulting in only moderate balanced accuracies of 65.4%. Similarly, Polyphen2_HDIV exhibited relatively balanced sensitivity (69.2%) and specificity (52.9%); a low balanced accuracy was similarly obtained (61.1%). A number of algorithms also exhibited a tendency to predict near‐all variants to be benign, which reduced discriminatory power; MutPred2 exhibited the extreme with 0% sensitivity, 100% specificity, and only a 50% balanced accuracy. MetaSVM and MetaLR displayed similarly stark prediction bias with 7.7% sensitivity, leading to balanced accuracies of only 53.8%, and PrimateAI similarly has low sensitivity (7.7%), producing a balanced accuracy of only 50.9%. This conservativeness in prediction may be partially a result of these algorithms’ training regimes, which emphasize minimizing FP predictions over true pathogenic variant detection.

**TABLE 2:** Performance of in silico tools before filtering for hemizygous variants in the population.

Software	TP	TN	FP	FN	Accuracy	Balanced accuracy	Sensitivity	Specificity	PPV	NPV	MCC	*F*‐score
SIFT	12	11	6	1	0.7667	0.7851	0.9231	0.6471	0.6667	0.9167	0.5767	0.7742
SIFT4G	9	14	3	4	0.7667	0.7579	0.6923	0.8235	0.75	0.7778	0.5218	0.72
Polyphen2_HDIV	9	9	8	4	0.6	0.6109	0.6923	0.5294	0.5294	0.6923	0.2217	0.6
Polyphen2_HVAR	5	14	3	8	0.6333	0.6041	0.3846	0.8235	0.625	0.6364	0.2332	0.4762
MutationTaster	4	17	0	9	0.7	0.6538	0.3077	1	1	0.6538	0.4485	0.4706
MutationAssessor	10	7	10	3	0.5667	0.5905	0.7692	0.4118	0.5	0.7	0.1903	0.6061
PROVEAN	5	13	4	8	0.6	0.5747	0.3846	0.7647	0.5556	0.619	0.1615	0.4545
MetaSVM	1	17	0	12	0.6	0.5385	0.0769	1	1	0.5862	0.2124	0.1429
MetaLR	1	17	0	12	0.6	0.5385	0.0769	1	1	0.5862	0.2124	0.1429
MetaRNN	9	14	3	4	0.7667	0.7579	0.6923	0.8235	0.75	0.7778	0.5218	0.72
M‐CAP	11	8	5	1	0.76	0.766	0.9167	0.6154	0.6875	0.8889	0.5538	0.7857
MutPred2	0	17	0	13	0.5667	0.5	0	1		0.5667		0
PrimateAI	1	16	1	12	0.5667	0.509	0.0769	0.9412	0.5	0.5714	0.036	0.1333
DEOGEN2	8	13	4	5	0.7	0.69	0.6154	0.7647	0.6667	0.7222	0.3845	0.64
BayesDel_addAF	4	17	0	9	0.7	0.6538	0.3077	1	1	0.6538	0.4485	0.4706
BayesDel_noAF	4	16	1	9	0.6667	0.6244	0.3077	0.9412	0.8	0.64	0.3309	0.4444
ClinPred	7	16	1	6	0.7667	0.7398	0.5385	0.9412	0.875	0.7273	0.5375	0.6667
LIST‐S2	5	12	5	8	0.5667	0.5452	0.3846	0.7059	0.5	0.6	0.0951	0.4348
ESM1b	4	17	0	9	0.7	0.6538	0.3077	1	1	0.6538	0.4485	0.4706
AlphaMissense	3	16	1	10	0.6333	0.586	0.2308	0.9412	0.75	0.6154	0.2507	0.3529

Abbreviations: FN, false negatives; FP, false positives; MCC, Matthews correlation coefficient; NPV, negative predictive value; PPV, positive predictive value; TN, true negatives; TP, true positives.

**FIGURE 6:**
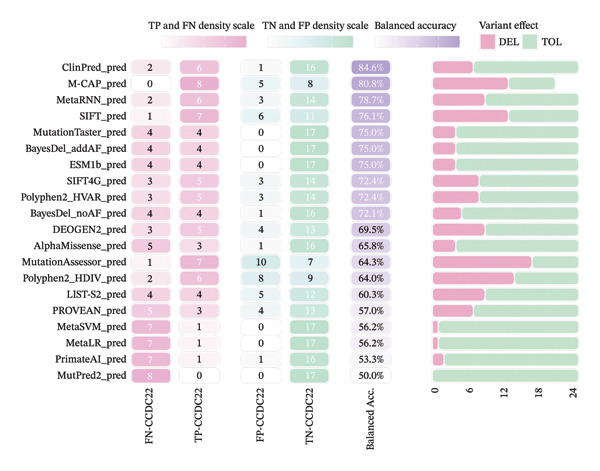
Performance evaluation of algorithms after filtering for hemizygous variants in the population. For each tool, the left heat‐map panel reports the rates of true positives (TP) and false negatives (FN) for disease‐associated variants and true negatives (TN) and false positives (FP) for control variants. The middle column gives the resulting balanced accuracy (balanced acc.), which was used to rank the tools from top to bottom. The right stacked bar shows the proportion of variants each algorithm called deleterious (DEL, red) or tolerated (TOL, blue) using recommended thresholds.

After filtering for hemizygous variants in the population, ClinPred showed the highest balanced accuracy of 84.6%, demonstrating substantial improvement in overall classification performance (Figure 6, Table 3). M‐CAP ranked second with 80.8% balanced accuracy, maintaining its robust predictive capability. MetaRNN achieved 78.7% balanced accuracy, while SIFT showed 76.1% balanced accuracy. Several tools that previously exhibited imbalanced performance metrics demonstrated improved balance after filtering. MutationTaster, BayesDel_addAF, and ESM1b all achieved 75.0% balanced accuracy with enhanced sensitivity (50.0%) while maintaining perfect specificity (100%). Polyphen2_HVAR showed marked improvement, reaching 72.4% balanced accuracy with balanced sensitivity (62.5%) and specificity (82.4%). However, the prediction bias observed in certain algorithms persisted even after filtering. MutPred2 continued to exhibit 0% sensitivity and 100% specificity, maintaining its 50.0% balanced accuracy. MetaSVM and MetaLR showed only marginal improvement with sensitivities of 12.5% and balanced accuracies of 56.2%. MutationAssessor displayed inconsistent performance with high sensitivity (87.5%) but low specificity (41.2%), resulting in a balanced accuracy of 64.3%.

**TABLE 3:** Performance of in silico tools after filtering for hemizygous variants in the population.

Software	TP	TN	FP	FN	Accuracy	Balanced accuracy	Sensitivity	Specificity	PPV	NPV	MCC	*F*‐score
SIFT	7	11	6	1	0.72	0.761	0.875	0.6471	0.5385	0.9167	0.4874	0.6667
SIFT4G	5	14	3	3	0.76	0.7243	0.625	0.8235	0.625	0.8235	0.4485	0.625
Polyphen2_HDIV	6	9	8	2	0.6	0.6397	0.75	0.5294	0.4286	0.8182	0.2626	0.5455
Polyphen2_HVAR	5	14	3	3	0.76	0.7243	0.625	0.8235	0.625	0.8235	0.4485	0.625
MutationTaster	4	17	0	4	0.84	0.75	0.5	1	1	0.8095	0.6362	0.6667
MutationAssessor	7	7	10	1	0.56	0.6434	0.875	0.4118	0.4118	0.875	0.2868	0.56
PROVEAN	3	13	4	5	0.64	0.5699	0.375	0.7647	0.4286	0.7222	0.1451	0.4
MetaSVM	1	17	0	7	0.72	0.5625	0.125	1	1	0.7083	0.2976	0.2222
MetaLR	1	17	0	7	0.72	0.5625	0.125	1	1	0.7083	0.2976	0.2222
MetaRNN	6	14	3	2	0.8	0.7868	0.75	0.8235	0.6667	0.875	0.5574	0.7059
M‐CAP	8	8	5	0	0.7619	0.8077	1	0.6154	0.6154	1	0.6154	0.7619
MutPred2	0	17	0	8	0.68	0.5	0	1		0.68		0
PrimateAI	1	16	1	7	0.68	0.5331	0.125	0.9412	0.5	0.6957	0.1138	0.2
DEOGEN2	5	13	4	3	0.72	0.6949	0.625	0.7647	0.5556	0.8125	0.3787	0.5882
BayesDel_addAF	4	17	0	4	0.84	0.75	0.5	1	1	0.8095	0.6362	0.6667
BayesDel_noAF	4	16	1	4	0.8	0.7206	0.5	0.9412	0.8	0.8	0.5145	0.6154
ClinPred	6	16	1	2	0.88	0.8456	0.75	0.9412	0.8571	0.8889	0.7181	0.8
LIST‐S2	4	12	5	4	0.64	0.6029	0.5	0.7059	0.4444	0.75	0.2001	0.4706
ESM1b	4	17	0	4	0.84	0.75	0.5	1	1	0.8095	0.6362	0.6667
AlphaMissense	3	16	1	5	0.76	0.6581	0.375	0.9412	0.75	0.7619	0.4023	0.5

Abbreviations: FN, false negatives; FP, false positives; MCC, Matthews correlation coefficient; NPV, negative predictive value; PPV, positive predictive value; TN, true negatives; TP, true positives.

**FIGURE 7:**
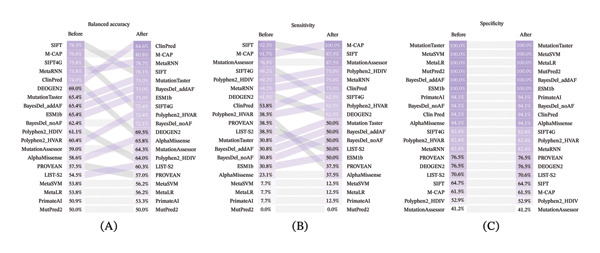
Comparison of performance metrics for 20 in silico prediction tools. The changes in predictive performance for *CCDC22* missense variants before/after filtered for hemizygous variants in the population were shown. The panels display the rankings and scores for (A) balanced accuracy, (B) sensitivity, and (C) specificity. In each column, tools are ordered in descending order based on their performance values (percentages). Lines connect the same tool across the two conditions to visualize shifts in rankings and metric scores. Darker purple indicates higher performance scores, while lighter shades represent lower scores.

Comparative analysis of the three key performance metrics before and after gnomAD filtering revealed differential impacts across prediction algorithms (Figure 7). The majority of tools showed improvement or maintenance of balanced accuracy following filtering, with ClinPred demonstrating the most substantial gain from 74.0% to 84.6%. Sensitivity changes were particularly pronounced in several algorithms: M‐CAP achieved 100% sensitivity after filtering compared to 91.7% before, while MutationAssessor increased from 76.9% to 87.5%. Conversely, some tools experienced sensitivity reductions. Specificity metrics generally remained stable or showed modest improvements across most algorithms, with MutationTaster, BayesDel_addAF, ESM1b, MetaSVM, MetaLR, and MutPred2 consistently maintaining 100% specificity both before and after filtering. These findings indicate that gnomAD filtering effectively refined the variant dataset and enhanced the discriminative performance of most prediction tools, particularly those employing ensemble learning or integrated scoring frameworks, while algorithms with inherent prediction biases remained largely unaffected by the filtering strategy.

### 3.6. ROC Curves for the *In Silico* Tools

**FIGURE 8:**
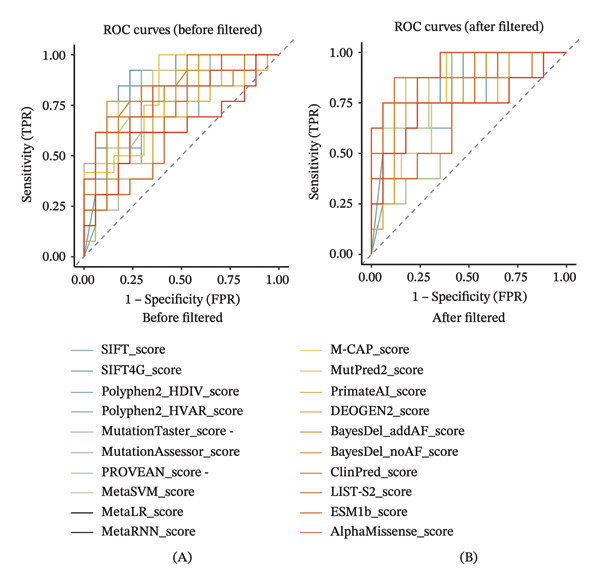
Receiver operating characteristic curve (ROC) performance and optimal thresholds of in silico tools for *CCDC22* variants. Combined ROC curves showed each color is one tool, and the dashed line meant no discrimination. All curves are provided in Figure 9.

**FIGURE 9:**
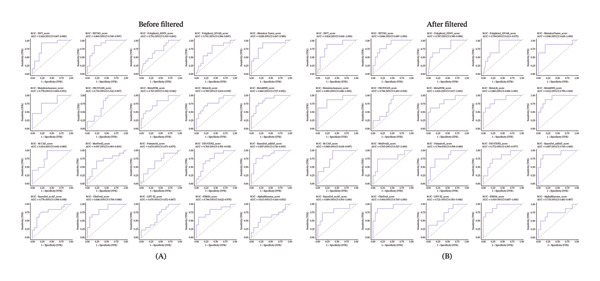
All ROC curves, including the unfiltered dataset and the filtered dataset. ROC curves were presented with AUC and 95% CI, including SIFT, SIFT4G, Polyphen2_HDIV, Polyphen2_HVAR, MutationTaster, MutationAssessor, PROVEAN, MetaSVM, MetaLR, MetaRNN, M‐CAP, MutPred2, PrimateAI, DEOGEN2, BayesDel_addAF, BayesDel_noAF, ClinPred, LIST‐S2, ESM1b, and AlphaMissense.

To visualize and evaluate the predictive performance of the tools, we generated ROC curves for all 20 prediction algorithms. AUC is a quantitative measure based on the ROC curve that reflects the overall diagnostic accuracy of a tool for all possible classification thresholds. Prior to applying the gnomAD filtering approach, ROC curve analysis revealed considerable variability in discriminative performance between algorithms (Figures 8A and 9A). ClinPred is the top‐performing tool with an AUC value of 0.917, followed by SIFT4G and MetaRNN (both 0.904), MutationTaster, BayesDel_addAF, and ESM1b (all 0.863), M‐CAP (0.858), BayesDel_noAF (0.843), SIFT and DEOGEN2 (both 0.824), AlphaMissense (0.810), Polyphen2_HVAR (0.796), PROVEAN, MetaSVM, MetaLR, and MutPred2 (all 0.747), MutationAssessor (0.746), PrimateAI (0.744), Polyphen2_HDIV (0.741), and LIST‐S2 (0.714). The AUC scores suggest that ensemble‐based tools such as ClinPred, MetaRNN and SIFT4G showed relatively better capacity to discriminate pathogenic variants from benign compared to other algorithms.

After filtering out variants observed as hemizygotes in gnomAD, we see increased ROC curve performance for most prediction algorithms (Figures 8B and 9B). ClinPred remained the top performer with an impressive 0.963 AUC, followed by M‐CAP (0.946), MetaRNN (0.919), SIFT4G (0.897), Polyphen2_HVAR (0.897), SIFT (0.893), MutationTaster, BayesDel_addAF, and ESM1b (all 0.886), BayesDel_noAF (0.885), DEOGEN2 (0.874), AlphaMissense (0.825), MutationAssessor (0.804), Polyphen2_HDIV (0.797), LIST‐S2 (0.751), PrimateAI (0.733), and PROVEAN, MetaSVM, and MetaLR (all 0.729). The filtering strategy resulted in marked AUC improvements for a few tools with M‐CAP going from 0.858 to 0.946, SIFT from 0.824 to 0.893, and Polyphen2_HVAR from 0.796 to 0.897, suggesting that the removal of common population variants has substantially boosted their discriminative power.

## 4. Discussion

In this study, we analyzed a multilevel biological context‐informed view of *CCDC22* and assessed twenty in silico algorithms for the prediction of the pathogenicity of missense *CCDC22* variants using both an unfiltered and a population hemizygote‐filtered dataset. This study summarized the *CCDC22* variants. We find ensemble‐based approaches, specifically ClinPred and MetaRNN, outperformed the other individual predictors across several fronts (sensitivity, specificity, MCC, and AUC). Filtering out variants that are seen as hemizygotes in the general population (such as gnomAD) may improve the majority of the tested tools. The results illustrate that integrating spatiotemporal expression, protein interaction networks, and population‐stratified filtering with computational predictions may improve the genetic diagnosis.

This study provided a biological context‐informed classification of *CCDC22* variants. The spatiotemporal expression pattern analysis revealed that *CCDC22* is ubiquitously expressed across adult tissues, with high expression in fetal development and late‐stage cortical neurogenesis, suggesting its critical role in neuronal differentiation and maturation. This establishes a “pathogenicity window” where variants should be more heavily weighted when identified in patients with neurodevelopmental phenotypes aligning with these critical developmental stages. Furthermore, the PPI network positions *CCDC22* as a central regulator of endocytic recycling and vesicle‐mediated protein sorting. Specifically, *CCDC22* acts within the CCC complex, interacting with known NDD‐related proteins like *VPS35L*. This biological context suggests that missense variants located within domains that mediate these specific PPIs, such as the coiled‐coil domains, are more likely to disrupt essential cellular trafficking and lead to disease. Incorporating this gene‐specific biological context‐informed approach allows clinicians to better genetic diagnosis.

The high performance of ClinPred and MetaRNN can be attributed to the meta design. Rather than applying naïve mapping solely to evolutionary conservation or some protein feature, these top classifiers stood apart as tools that were built using ensemble learning frameworks that capture multidimensional data. ClinPred incorporates gnomAD frequency information and is trained on ClinVar datasets, likely bringing its underlying predictive logic more in line with pathological classification as of today. MetaRNN’s use of deep learning to combine genomic annotations captures more complex, nonlinear associations between sequence variation and pathogenicity. This may partly explain why their predictions remained stable and discriminative (AUC > 0.9), as opposed to MutPred2 or MetaSVM, which showed significant bias toward sensitivity or specificity in the context of *CCDC22*. However, our ROC analysis revealed that their underlying discriminative capacities are not inherently poor; their AUC scores were moderate and improved after filtering; MutPred2: 0.697–0.765). This stark discrepancy between 0% sensitivity and moderate AUC values indicates that the perceived underperformance is an issue of threshold calibration. The generalized pathogenicity thresholds of these tools appear overly stringent or poorly calibrated for *CCDC22* missense variants. While calculating gene‐specific thresholds could potentially rescue their sensitivity, our currently limited sample size precludes such recalibration without a high risk of overfitting.

This study revealed how susceptible the algorithms are to “noise” in training or testing data; tools M‐CAP, Polyphen2_HVAR, and SIFT show dramatic improvements to AUC and balanced accuracy after the exclusion of population hemizygotes, with M‐CAP’s sensitivity increasing to 100% and AUC score increasing substantially, suggesting they are very good at discovering true pathogenic variants but are easily confused by variants which in silico appear damaging but which are present in healthy populations. The statistic’s further difference in tools like Polyphen2, which does not become significant until after filtering, suggests how the presence of confusing variants (which exhibit conflicting computational scores and population behavior) degrades the true performance of predictive software.

A complex issue addressed in this study is the presence of reported “pathogenic” variants in the gnomAD database as hemizygotes. For a gene like *CCDC22*, which is associated with severe neurodevelopmental phenotypes such as RTSC2 and X‐linked epilepsy, causative variants should not be observed in hemizygous states in a healthy male population. However, the occurrence of these variants in controls suggests several possibilities: incomplete penetrance, variable expressivity, or the existence of subclinical phenotypes. It is plausible that carriers in the general population may manifest mild or nonclinical features—such as folliculitis, alopecia, or other subtle traits—that do not significantly impair daily life and thus escape strict clinical diagnosis. Consequently, while the severe syndromic presentation of *CCDC22* mutations supports a strong gene‐disease association, the overlap with population controls cannot be dismissed. These findings suggest that while the association is valid, specific variants require rigorous re‐evaluation to avoid misdiagnosis, and further phenotyping of “healthy” carriers would be invaluable.

The fact that prediction accuracy was improved for nearly all software if variants present as hemizygotes were removed is a compelling argument for a “population‐first” filtering approach. The fact that the accuracy improved on the filtered dataset showed that variants present as hemizygotes in large control databases are likely to be either benign or of low penetrance. This yields a macroscopic view of variant interpretation: the biological fact of survival and reproduction in the population (as evidenced by hemizygosity) acts as a major filter that trumps in silico predictions. Thus, evaluating whether a variant is present in population databases should be considered not just a frequency check but rather validation that decreases FPs when combined with high‐performing tools like ClinPred.

Despite the promising results, with ClinPred achieving a balanced accuracy of 84.6% and an AUC of 0.963, there remains room for improvement. Disorders caused by *CCDC22*‐related variants encompass a broad phenotypic spectrum—from isolated (pure) intellectual disability through to complex syndromic presentations concurrent with dyslipidemia and cerebellar malformations. The fact that not a single tool was able to generate perfect prediction parameters reinforces the difficulty of modeling phenotypic heterogeneity computationally, where current tools employ a binary classification of disease‐associated versus benign—potentially failing to incorporate nuances from pleiotropic genes. While current software to predict the pathogenicity of novel variants maintains an essential clinical utility, there is an unmet need for next‐generation tools that incorporate specific phenotype–genotype correlations, and these would add significant precision in making a diagnosis for genes with clinically wide spectrums.

Missense variants, a major component of disease‐causative variants, are the most prevalent type identified in clinical practice. Our recent investigations have indicated that missense variants are frequently implicated in epilepsy when occurring in well‐characterized NDD‐associated genes, such as *ACTB* [32], *DLG3* [33], *EP400* [34], *GABRA1* [35], *GALC* [36], *SRCAP* [37], *MACF1* [38], *SZT2* [39], *TANC2* [40], and *KCNK4* [41]. This study highlights the continuing need to develop in silico pathogenicity prediction tools. As the pace of genetic testing continues to increase, algorithms to support clinical decision‐making will become essential. Similar to our previous research [20, 21], the integration of gene‐specific functional datasets with carefully curated clinical evidence is a practical strategy to evaluate and further develop gene‐specific predictors with a limited amount of bias.

Several limitations in this study should be acknowledged. Firstly, the limited sample size of curated missense variants causes highly volatile performance metrics, where a single reclassified variant can shift the ranked order of all evaluated tools. Therefore, the reported estimates should be treated as exploratory baselines rather than reliable indicators of generalizable performance. Secondly, the absence of an independent or external validation dataset may cause a risk of overfitting. The findings regarding algorithmic performance and population‐based filtering benefits should not be directly be extrapolated to other X‐linked genes without validation in larger, independent cohorts. Future research should prioritize expanding variant datasets, integrating functional assays, and re‐evaluating variant classifications.

In summary, this study is a systematic gene‐specific evaluation of 20 in silico tools for predicting *CCDC22* missense variant pathogenicity. ClinPred and MetaRNN showed potentially higher predictive potential, achieving balanced accuracy rates of 84.6% and 78.7% (AUC 0.963 and 0.919) after gnomAD hemizygous variant filtering; this population‐based approach improved the robustness of most tools by minimizing confounding factors from benign/low‐penetrance variants. These results may bridge a clinical gap of evidence‐based *CCDC22* variant classification, improve diagnostic yield for related disorders, and provide an approach for annotating other X‐linked genes.

## Author Contributions

Conceptualization, P‐Q.Z., P‐Y.W., S‐Q. Z., H.M., H.L., J.G., and S.L.; methodology, P‐Q.Z., P‐Y.W., and S‐Q. Z.; software, S‐Q. Z. and P‐Y.W; writing–original draft preparation, P‐Q.Z., Y.W., and S‐Q. Z.; writing–review and editing, P‐Q.Z., P‐Y.W., Y.D., W‐H. L., K.P., H.M., H.L., J.G., and S‐Q. Z.; visualization, P‐Y.W.

## Funding

This research was funded by the Medical Scientific Research Foundation of Guangdong Province, China, grant number A2024536.

## Disclosure

All authors have read and agreed to the published version of the manuscript.

## Ethics Statement

This research study did not involve any human participants or animal experiments. Therefore, no ethical approval was required, and no ethical issues were raised during the course of this research. We adhered to all relevant laws and regulations regarding research and data usage.

## Conflicts of Interest

The authors declare no conflicts of interest.

## Supporting Information

Additional supporting information can be found online in the Supporting Information section.

## Supporting information


**Supporting Information** Table S1. Software included in this study.

## Data Availability

Data are available from the corresponding author upon reasonable request.
