# Supplementary material for: Biological Context–Informed and Population‐Stratified Strategies Improve Genetic Diagnosis of CCDC22‐Related Disorder
Source: Genet Res (Camb). 2026 Jun 24;2026:8137770. doi: 10.1155/genr/8137770 (PMC13292020; doi:10.1155/genr/8137770)
Supplement: Supplementary file 1 — Supporting Information Table S1. Software included in this study. [file GENR-2026-8137770-s001.docx]

**Table S1. Software included in this study**

| **Software** | **Description** | **Pathogenic cutoff** |
| --- | --- | --- |
| SIFT | A tool utilizes sequence conservation and amino acid properties to predict the impact of amino acid substitutions on protein function[1]. | <0.05 |
| SIFT 4G | A faster version of SIFT, capable of providing predictions for a large number of organisms [2]. | <0.05 |
| PolyPhen-2_HDIV | The HumDiv-trained version (HDIV) is optimized for assessing rare alleles potentially involved in complex traits, fine-mapping regions identified through genome-wide association studies, and evolutionary analyses where even mildly deleterious variants are considered damaging[3]. | >0.5 |
| PolyPhen-2_HVAR | The HumVar-trained version (HVAR) is specifically designed for the diagnosis of Mendelian diseases, aiming to distinguish strongly deleterious mutations from the remaining human variation, including the presence of mildly damaging alleles[3]. | >0.5 |
| Mutation Assessor | A tool predicts functional impact based on the evolutionary conservation of affected amino acids in protein homologs[4]. | >1.935 |
| PROVEAN | A tool combines evolutionary conservation, neural network models, and the BLOSUM62 scoring matrix to assess variant impact [5]. | <-2.5 |
| MetaSVM | An ensemble-based predictor for missense variant deleteriousness. It integrates nine individual deleterious prediction scores along with the maximum minor allele frequency[6]. | >0 |
| MetaLR | An ensemble scoring method for deleterious missense mutations, which demonstrated the value of combining information from multiple orthologous approaches[6]. | >0.5 |
| MetaRNN | A deep recurrent neural network–based ensemble models that integrate 28 high-level annotation features, including multiple functional prediction scores, evolutionary conservation metrics, and allele frequency information, to predict the pathogenic likelihood of human nonsynonymous SNVs and non-frameshift indels[7]. | >0.5 |
| M-CAP | A pathogenicity classifier for rare missense variants that integrates existing prediction scores and additional genomic features within a high-sensitivity model, optimized for clinical use [8]. | >0.025 |
| MutPred2 | A machine learning–based predictor that employs an ensemble of neural networks trained on large sets of pathogenic and putatively neutral variants [9]. | >0.737 |
| PrimateAI | A tool uses deep neural networks to predict the clinical impact of human mutations, incorporating primary sequence and protein structure information [10]. | >0.803 |
| DEOGEN2 | Integrates heterogeneous information about molecular effects, domains, gene relevance, and protein interactions to predict variant deleteriousness [11]. | >0.5 |
| BayesDel_addAF/ BayesDel_noAF | A Bayesian ensemble-based meta-predictor that estimates the deleteriousness of both coding and non-coding variants, including single-nucleotide variants and small insertions or deletions[12]. | >0.0692655/ >-0.0570105 |
| ClinPred | A machine learning–based predictor for disease-associated missense variants that integrates existing pathogenicity scores with population allele frequency from gnomAD database and is trained on ClinVar data to achieve highly accurate and robust pathogenicity classification across diverse disease contexts[13]. | >0.5 |
| LIST-S2 | Successor to LIST, quantifies conservation across species and predicts variant deleteriousness, not limited to human sequences [14]. | >0.85 |
| ESM1b | A 650‑million‑parameter protein language model trained on 250 million protein sequences from diverse organisms using a masked language modeling objective, in which randomly masked residues are predicted based on their surrounding sequence context [15]. | <-7.5 |
| AlphaMissense | An adaptation of AlphaFold, which trained on databases of population frequencies of human and primate variants, incorporating structural context and evolutionary conservation as parameters for predicting missense variant pathogenicity [16]. | >0.564 |
| MutationTaster | A tool employs a Bayes classifier to eventually predict pathogenicity, which is fed with the outcome of all tests and the features of the alterations and calculates probabilities for the alteration to be either a disease mutation or a harmless polymorphism[17]. | >0.5 |

**Reference**

1. Kumar, P., S. Henikoff, and P.C. Ng, *Predicting the effects of coding non-synonymous variants on protein function using the SIFT algorithm.* Nat Protoc, 2009. **4**(7): p. 1073–81.

2. Vaser, R., et al., *SIFT missense predictions for genomes.* Nat Protoc, 2016. **11**(1): p. 1–9.

3. Adzhubei, I.A., et al., *A method and server for predicting damaging missense mutations.* Nat Methods, 2010. **7**(4): p. 248–9.

4. Reva, B., Y. Antipin, and C. Sander, *Predicting the functional impact of protein mutations: application to cancer genomics.* Nucleic Acids Res, 2011. **39**(17): p. e118.

5. Choi, Y., et al., *Predicting the functional effect of amino acid substitutions and indels.* PLoS One, 2012. **7**(10): p. e46688.

6. Dong, C., et al., *Comparison and integration of deleteriousness prediction methods for nonsynonymous SNVs in whole exome sequencing studies.* Hum Mol Genet, 2015. **24**(8): p. 2125–37.

7. Li, C., et al., *MetaRNN: Differentiating Rare Pathogenic and Rare Benign Missense SNVs and InDels Using Deep Learning.* bioRxiv, 2021: p. 2021.04.09.438706.

8. Jagadeesh, K.A., et al., *M-CAP eliminates a majority of variants of uncertain significance in clinical exomes at high sensitivity.* Nature Genetics, 2016. **48**(12): p. 1581–1586.

9. Pejaver, V., et al., *Inferring the molecular and phenotypic impact of amino acid variants with MutPred2.* Nature Communications, 2020. **11**(1): p. 5918.

10. Sundaram, L., et al., *Predicting the clinical impact of human mutation with deep neural networks.* Nat Genet, 2018. **50**(8): p. 1161–1170.

11. Raimondi, D., et al., *DEOGEN2: prediction and interactive visualization of single amino acid variant deleteriousness in human proteins.* Nucleic Acids Res, 2017. **45**(W1): p. W201–W206.

12. Pejaver, V., et al., *Calibration of computational tools for missense variant pathogenicity classification and ClinGen recommendations for PP3/BP4 criteria.* Am J Hum Genet, 2022. **109**(12): p. 2163–2177.

13. Alirezaie, N., et al., *ClinPred: Prediction Tool to Identify Disease-Relevant Nonsynonymous Single-Nucleotide Variants.* Am J Hum Genet, 2018. **103**(4): p. 474–483.

14. Malhis, N., et al., *LIST-S2: taxonomy based sorting of deleterious missense mutations across species.* Nucleic Acids Res, 2020. **48**(W1): p. W154–W161.

15. Brandes, N., et al., *Genome-wide prediction of disease variant effects with a deep protein language model.* Nature Genetics, 2023. **55**(9): p. 1512–1522.

16. Cheng, J., et al., *Accurate proteome-wide missense variant effect prediction with AlphaMissense.* Science, 2023. **381**(6664): p. eadg7492.

17. Schwarz, J.M., et al., *MutationTaster2: mutation prediction for the deep-sequencing age.* Nature Methods, 2014. **11**(4): p. 361–362.
